# The paradigm model of distorted doctor-patient relationship in Southern Iran: a grounded theory study

**Published:** 2016-04-23

**Authors:** Ahmad Kalateh Sadati, Seyed Ziauddin Tabei, Najme Ebrahimzade, Mohsen Zohri, Hossein Argasi, Kamran Bagheri Lankarani

**Affiliations:** 1 Assistant Professor, Department of Sociology, Yazd University, Yazd, Iran; and Health Policy Research Center, Shiraz University of Medical Sciences, Shiraz, Iran;; 2 Professor, Department of Medical Ethics and Philosophy of Health, Shiraz University of Medical Sciences, Shiraz, Iran;; 3 Health Policy Research Center, Shiraz University of Medical Sciences, Shiraz, Iran;; 4 Department of Sociology, Shiraz University, Shiraz, Iran;; 5 Research Center Consultation (RCC), Shiraz University of Medical Sciences, Shiraz, Iran;; 6 Professor, Health Policy Research Center, Shiraz University of Medical Sciences, Shiraz, Iran.

**Keywords:** *Doctor-patient relationship*, *Patient satisfaction*, *Paradigm model*, *Iran*

## Abstract

The doctor-patient relationship (DPR) is one of the most important subjects in medical sociology and health policy. Due to mutual understanding, undistorted DPRs not only result in satisfaction of both doctors and patients, but also help to reduce financial burdens for patients and the health care system. The purpose of this research was to identify a DPR based on the qualitative paradigm model which is called the grounded theory (GT) methodology. The data were collected from 3 focus groups, the participants of which consisted of 21 faculty members of Shiraz University of Medical Sciences, Shiraz, Iran. The content of the interviews, following the transcription stage, was organized based on open, axial, and selective coding. Results showed that DPR was *distorted* which was the consequence of an *inefficient structure* in the healthcare system which is related to several *cultural barriers*. In this situation, agency is determinant so the *doctor's personality* determines the direction of DPR. Consequences of such scenarios are the *patient’s distrust*,* patient's dissatisfaction*,* lack of mutual understanding*, *patient suppression*,* and patient deception*. Therefore, the health care system should emphasize on reforming its inefficient infrastructures, so that, besides being controlled and surveyed, physicians are socialized ethically.

## Introduction

The doctor-patient relationship (DPR) is the main subject of medical ethics, health policy and management. The doctor-patient relationship (DPR) is the keystone of care (1). Its meaning, although seemingly simple, is complex (2) and poorly defined (3), and has uncertain features (4, 5). Although the debate on DPR began in Hippocrates’ era, it has remained a central topic in medical discourse over the past decades (6, 7). DPR is a controversial topic because it is an interaction between two human beings with distinct values and characteristics. A dynamic and mutual relationship not only can lead to satisfaction of both parties (8-11), but also helps to reduce financial burden for patients and the health care system. Moreover, DPR is an important topic in medical ethics (12). From this perspective, DPR is not simply a matter of professionalism, but it has philosophical dimensions (13).

Since the 1950s, two different sociological paradigms have addressed DPR (6, 14). On the one hand, Parsons’ functionalist theory (15) has concentrated on the patient’s role, recognizing 4 roles as the patient’s responsibilities toward his/her illness and toward doctors’ medical instructions (16). On the other hand, certain critical theories have been proposed including Habermas’ critique on modern specialization (17, 18), and criticisms on knowledge-power relations established by modern medicine from Foucault’s viewpoint (19). In addition, there have been extensive ethnographic studies criticizing modern medicine (20, 21). 

Due to the importance of DPR, it has been studied in different disciplines such as psychiatry (22), dermatology (23), somatic symptom severity (SSS) (24), cerebral aneurysm surgery (25), pharmaceutical care (26), and chronic illness (27). While good DPR can lead to better clinical outcomes (2, 28) and patients' satisfaction, poor DPR can negatively affect quality of healthcare services (2). Therefore the purpose of this study was to identify the quality of DPR, based on the views of Shiraz University of Medical Sciences faculty members. To achieve this goal, this study tried to explore paradigm model of the DPR; a model which is capable of showing phenomenon, context, intervening conditions, and action/interaction strategies to consequences. This is an inductive study which explores the codes in three phases and a paradigm model according to grounded theory method. 

## Method

This qualitative study was conducted between April and September 2014, among faculty members of Shiraz University of Medical Sciences. To maximize participation and collect different viewpoints (29), the focus group discussions (FGDs) method was applied. During each interview, at least 6 faculty members were present and the interviews were recorded digitally after obtaining a verbal consent. Data were transcribed after each discussion. Data collection and theme analysis were performed simultaneously. When data saturation was achieved (30, 31), sampling finalized. Our method was grounded theory included two main phases: exploring codes in three phases and presenting findings in paradigm model. 

Initially, coding process was conducted in three main phases: open, axial and selective coding. Open coding is the first stage of analysis as well as comprehensive analysis. According to Glaser, open coding is the initial stage of comparative analysis. At this stage, analysis was performed through a line-by-line analysis of data. There are many data coding methods including writing memos about the conceptual and theoretical ideas that emerged throughout the analysis (32). In this stage we interpreted the meaning units. Axial coding was the second stage. The purpose of axial coding is to put the fractured data together in new ways “by making connections between a category and its subcategory” (33). During axial coding, we tried to understand categories observed in relation to other categories and their subcategories (34). Based on axial coding we tried to explore the phrasal code which presented the phenomena in higher level of abstraction. At the third stage, called selective coding, data were integrated to shape a central theme, hypothesis, or story to generate a theory (35). Selective coding is the highest abstraction of coding. In this level code is explored according to meaning units, and other previous codes and also based on previous theoretical frameworks about the subject. One practical sample of coding in three phases about code called distorted DPR is showed in table 1.

After exploring the codes, another important part of the study was presenting findings in a paradigm model based one Strauss and Corbin’s (32) approach to GT. Based on Strauss and Corbin’s approach, a paradigm model should distinguish between causal conditions and intervening conditions and there is a very strict linear model for causal conditions via phenomenon, context, intervening conditions, and action/interaction strategies to consequences. After the analysis of data, differences between them were found using the paradigm model. For the presentation of the paradigm model, we used theoretical sampling through inductive and deductive approaches. To this end, we developed through with a reflexive comparative analysis of data and codes for a better understanding of the theory produced. 

Since, this article was based on a sociological study, to observe ethical principles of research, the codes of the American Sociological Association were applied throughout different stages of this research. The criterion sampling was finalized after data saturation was reached (34, 35). Research validation was conducted through member check.

**Table 1:** An example of three phases coding process about distorted DPR

Meaning units	Open codes	Axial codes	Selective code
There are several patients which you or your assistant do many works for them, but because you don’t have relationship with them, they don’t benefit from them or they don’t satisfy,	Patient deson’t understand doctors’ efforts.	Unappeasable patient	Distorted DPR
After public system, condition is worse, some physician work until 4 in the morning, because we don’t know what we want from this interaction.	Doctors just work without interaction.	Ignorant and irregular relationship.	
In our country there is a different form of relationship in comparison with other countries. Somehow the doctor believes himself to have the right to treat his patient any way he/she sees fit.	Relationship depends on doctor’s desires.		
One day a patient whose pathology results were positive for malignancy came to me with his wife. His wife worried and was about to faint. They asked me to tell them the facts, but I did not tell them everything. I asked them go to their doctor and just ignore them.	Doctors do not reply appropriately to patients	Ignorance of patent’s psychological need	
It has occurred numerous times; the patient does not communicate some main points of his/her illness, due to the doctor’s week interaction.	Patient does not present a complete history of his/her illness.	Incomplete relationship.	

## Results

DPR has had an important role in the treatment process, to such an extent that it is called “the key to treatment” (Surgeon of FGD1)). As a result of lack of good DPR, doctors fail to appropriately practice medicine. Yet, the DPR within the context of this study was specifically leaning towards doctors’ benefits, while patients and their concerns were disregarded. Under these circumstances, the conditions governing the consultations in clinics and hospitals did not allow for a suitable means of communication. This means that doctors practice medicine without establishing any kind of mutual interaction, but mainly a one-sided relationship. This type of interaction is called distorted DPR. 

**Distorted DPR**

Every relationship is initiated with interaction and dialogue. In contrast, a distorted relationship is one-sided. In the distorted relationship, little or no communication takes place between the doctor and patient. In such interactions, not only the scientific principles of diagnosis and treatment, but also meaningful action are disregarded. The consultation is conducted within a few minutes, and then, the patient leaves the office with only a written prescription. The participants’ statements showed that a distorted DRP, within the context under study, had turned into a norm and was based on doctors’ autonomy. 

[*Even today, there are still some physicians, even good ones, who do not even talk to their patient (Nephrologist of FGD3). In governmental hospitals, verbal communication has sharply declined. The situation is such that the doctor performs an examination, and then, makes a decision without any verbal communication.*] (Psychologist of FGD3) 

“Do not even talk” indicates a total absence of verbal contact between the doctor and patient. In such situations, diagnosis and treatment are mainly based on the doctor’s experiences or para-clinical data. When no dialogue takes place, no message is conveyed, and as a result, no meaning (patient’s feeling and experience) is transferred. In extreme cases, the doctor disregards the patient’s existence as a human being and the patient leaves the office feeling perplexed. A distorted relationship is a mechanical and passive one. Consequently, the doctor does not gain any understanding of the patient’s perception of and attitude toward the illness. 

[*A mechanical relationship means that no attention is paid to the patient’s psyche; the person in front of you is called a human being whose soul and psyche should also be taken into account. By considering his/her psychological issues, you can treat many of them. The patient’s psychological aspects should not be ignored or suppressed, and he/she should not be treated like an object. Treating a patient should not be like repairing a car. The doctor does not look at the patient, but looks at the lab test papers*.] (Psychologist of FGD2)

In other words, one-sided or mechanical relationships are generally unequal. There is even a higher level of inequality in doctors’ conduct and their patterns of questioning, shaping an interrogator-defendant-style dialogue.

[*Doctors are not supposed to act like interrogators. Some doctors are just like that and when a person sits in front of them, they start asking questions in this manner*.] (Psychiatrist of FGD1) 

In an interrogative style interaction, the patient faces numerous closed questions which should only be answered with yes or no. Yet, if the patient wishes to change the direction of the consultation by asking a question about the diagnosis or therapy, the question is simply suppressed. If the patient insists, he/she may receive an unpleasant answer. A distorted DPR is an interrogative interaction which includes weak, mechanical, and unequal communication. 

**Inefficient infrastructure**

Inefficient infrastructure was the chief issue observed in relation to the shaping of distorted relationships. One aspect of this issue is the inability to respond to a large number of patients, including clinical and hospitalized patients, due to poor accessibility. Doctors are forced to consult a great number of patients on a daily basis. 

[*When I have to visit 100 patients in the hospital, I cannot even understand what the thirtieth or fortieth patient says, or I simply refer him to my resident. How strong do you think I am to visit more than 100 patients!?*] (Nephrologists of FGD3) 

This statement shows that a doctor has limited capacity in providing services. For instance, a psychotherapist “has to spend at least thirty minutes on each patient” (Psychiatrist of FGD1), although those in charge of the clinic have a different expectation. 

[*The biggest obstacle is the system. In fact, our flawed health system forces the attending physicians to visit 50-60 patients a day, while visits are heavily dependent on interaction, and without it, even in case of emergency, no matter how much I try to help, it will not work. The clinic manager encourages me to visit 70 patients, but I need to spend at least 0:30 minutes on each visit. On the contrary, if I do this it has negative implications for me. It means that I am not a doctor, but more like a money-making machine.*] (Psychiatrist of FGD1) 

Poor accessibility to physicians has led the health system to focus on quantitative services rather than qualitative services. Meanwhile, a quantity-oriented service could affect the quality of consultations and relationships. The health system has focused on quantity not only in case of providing services, but also in case of science and knowledge. In such a condition, the criterion for professors’ promotion is simply the number of papers they have published rather than their performance. 

[*In professors’ promotion form, there is no item for quality, and the whole thing is about the number of papers published. The best professor I have seen with numerous papers allows half a dozen patients at a time in his office, and never lets them talk, because for his promotion, quality is not considered, and only the number of papers is important. I wonder how much these papers will help patients.*] (Internist of FGD2)

Since the criterion for doctors’ evaluation is the number of their papers and not the quality of their performance, they pay no attention to active interaction. Another characteristic of inefficient infrastructure is poor control/supervision, or even lack of supervision, which is a shortcoming that has direct impact on DPR. 

[*I just wanted to say that we are not satisfied ourselves; there is not even a system to put us within a framework, and this is a serious obstacle.*] (Surgeon of FGD1) 

When there is no framework and there are economic demands prompting the doctor to compete against other doctors, medicine is practiced without considering quality and active interaction with patients as a critical issue is simply marginalized. 

Another aspect of inefficient infrastructure is reflected upon one-dimensional education system, which creates one-dimensional medical logic. This education system encourages mechanical and instrumental relations. Such structures neglect to teach the ethics and philosophy of medicine. In this structure, DPR skills are not taught and ethical problems are not explored.

[*In our time, there was no course or workshop for medical ethics, and now, even at our residency stage, there still are no such courses! So, teaching such issues is not important and professors do not expect students to know about these things.*] (Psychiatrist of FGD2) 

Clearly, students neither receive medical ethics awareness nor communication skills training, and this framework reinforces distorted DPR, as a hidden curriculum.

[*When students come to Motahari Clinic to be trained, they learn how to interact from their professor. When they see a professor visits 70-80 patients per day, the students, too, learn this.*] [Pediatrician of FGD2] 

In addition to “hidden curriculum”, medical students are faced with the education/treatment paradox. This creates a struggle between educational departments and treatments.

[*Education and treatment are in conflict with each other. You sit in the department and see 4 protocols for the education of medicine with some orders. Then, you see 10 protocols for providing services to patients which are in conflict with the previous protocols.*] (Nephrologist of FGD3) 

Finally, inefficiency in the infrastructure can lead to inefficiency in the student admission screening process system which is performed without any protocols or plans.

[*At this point, medical students are not screened when they are entering medical colleges. Often, university graduates are even worse than the new entries (new students), because some of the students are not even cut out for the job*.] (Internist of FGD2) 

As a result, university graduates are not familiar with ethical principles; thus, in reality they cannot be expected to act accordingly.

**Cultural barriers**

Data revealed that a number of the themes in distorted relationships were in fact rooted in culture. One of the related cultural values is patient's obedience, which refers to patients’ attitudes that encourage them to yield to doctor’s dominance. This cultural attitude contributes to doctors’ imposition of power, and there seems to be a relationship between this attitude and doctors’ tendency for dominance. Apparently, acting congenially on the part of the doctor may convey the impression that he/she is not competent enough. 

[*In Iran, due to our culture or basically our Iranian personality, if a doctor allows us to have an active interaction with them during our visit, we feel that he/she does not have sufficient knowledge.*] (Surgeon of FGD1) 

One of the participants believed that this tendency was rooted in the cultural and social settings of Middle Eastern countries.

[*In Middle Eastern societies, patients still assume that a doctor must preserve and practice his authority.*] (Psychiatrist of FGD2) 

As a result, culture and cultural values demand from doctors to refrain from practicing the ethics of interaction. There is another side to this coin of cultural obedience; if a patient feels that the doctor has allowed him to communicate, the patient creatively tries to direct the dialogue towards his/her points of view. This is called patient's manipulation which is another cultural characteristic which can lead to the misuse of the doctor’s position by the patient. 

[*Some patients do not have the capacity to accept this courtesy and when you bring yourself to their level, they somehow try to manipulate you. If you want your patient to “buy” and cherish your words, you must create some sort of difference and you should talk from a higher status.*] (Anesthesiologist of FGD1) 

Doctor’s paternalism is another value that is reproduced in this context. Medical culture is itself a patriarchal and suppressive culture in which the doctor regards himself as “the guardian” of the patient. In this culture, doctors, as they think they deserve to practice medicine, regard themselves as experts in every aspect of treatment and disregard patients’ experiences.

[*A patriarchal medical culture is one in which the doctor considers himself as the custodian and somehow he allows himself to treat the patient as he wishes*.] (Dentist of FGD2) 

Another cultural issue is poor health literacy. The most challenging concerns are individuals’ attitudes toward illness and therapy, inability to present their illness and the inappropriate structure of communication with the doctor. 

[*Some patients come, let me tell you precisely, and I ask them, “What’s wrong with you?”, and in response they say, “How do I know! You’re the doctor here!” I then ask about their illness, but feeling shameful about their problem, such as rectal bleeding, they may say they have stomachache. They hide the truth and say something else instead.*] (Surgeon of FGD1) 

Another aspect of cultural barriers is related to the growth of materialistic doctors in Iran. Under such circumstances, a patient is disregarded as a human being in need of quality services and is viewed as a source of financial benefits. This perspective toward patients may, in some cases, encourage the doctor to deceive patients. 

[*The society is increasingly becoming materialistic and people are seen as dollar signs. This could be your child when he/she wants to go to private class with a teacher, a customer for a salesperson, the police, or a judge.*] (Ophthalmologist of FGD2) 

The more materialistic the relationship becomes, the more intensified the distorted DPR will become. This is because symbolic mediators turn to financial benefits instead of ethics and human dignity. Accordingly, doctors distance themselves from the ethics and philosophy of their profession and stay in limbo, hanging between human responsibility and materialistic benefits. 

[*The problem is that we do not know what we want from this relationship with our patient. Do we seek inner satisfaction, money, or social status? I know a person who makes $ 500.000 UDS per month and is still not satisfied. This physician has hired 10 surgeons as assistants to work for him and has told them they will not have any professional identity for the next 10 years, since they are operating under his name.*] (Surgeon of FGD1)

 Lack of openness to criticism and lack of freedom of opinion were other problems found in the context of the study, and they contribute to doctors' authoritative personality. Thus, doctors treat patients according to the cultural settings they were raised and educated in; that is, to be dominant over subordinates and to be submissive to their superordinates which generally disregard human dignity.

[*There is something called human dignity which in my opinion is genetic. Although it might seem insignificant, the whole story emerges from this point. To do this, a person should know what human dignity is, and this goes back to his/her childhood, the ages 1-6, and not necessarily the university system, where they have acquired their behavior.*] (Pediatrician of FGD3) 

The incomplete educational system leads to the formation of distorted DRP. In this educational culture, instead of an analytical qualitative outlook, a quantitative and test-achievement approach which overlooks other issues has become dominant. 

[*From secondary school, or lower education levels, students go to school to learn how to answer tests, but they do not learn much about thinking or communication. They only think about how to get good and better grades without any analytical approach. Finally, this person becomes a doctor who cannot communicate properly with his/her clients.*] (Pediatrician of FGD3)

***Consequences***

A distorted DPR with the above characteristics will have certain consequence in the interaction. The first outcome of incorrect diagnosis and distorted DPR is patients distrusting the doctor. 

[*Does the fact that patients should wait for a long period of time to visit a doctor lead to their mistrust or discouragement? Should they wander about for 2-3 months and follow wrong orders before you [doctor] may finally make a correct diagnosis?!*] (Surgeon of FGD1)

Patient's dissatisfaction with consultation and lack of mutual understanding are other consequences of distorted DPRs.

[*I can see in the eyes of some (patients), when they are leaving, that they think: “what a stupid idea it was to see this doctor; it’s as though I’m talking to a wall.” They feel that I do not understand them, or I feel they do not understand me.*] (Psychiatrist of FGD1)

Suppressing patients is yet another outcome of distorted DPR. The doctor fails to establish a good relationship with the patient and does not understand him/her, and thus, reprimands the patient’s attitude and action, suppressing them. In focus group 2, one of the psychotherapists retold a story of a gynecologist’s encounter with a patient, who due to illness had to have an abortion. 

[*First the doctor started out well and said that it was not a big deal and that only 1 out of 270 cases would need an abortion. At this point, the patient started to cry, to which the doctor responded offensively, asserting she had not said anything wrong and if the patient would not stop she had to leave.*] (Psychologist of FGD2) 

Deceiving patients is another consequence of DPR. Patients’ lack of adequate information about their health conditions is the cause of their concern and apprehension. Under such circumstances, the doctor can easily deceive the patient in order to acquire financial benefits. Some participants talked about “patient deception” in such fields as gynecology, ophthalmology, and dentistry.

[*The art of deception is so prevalent!!! For example, the patient is advised to do a transvaginal ultrasound test and without any examination, the doctor tells her that she has uterine prolapse and should be operated on right away. This is very prevalent in Iran. The patient starts crying, saying that the doctor told me, if I do not treat this issue, it may lead to cancer.*] (Gynecologist of FGD2)

One of the participants claimed that due to the high patient turnover in governmental hospitals, doctors are not motivated to participate in patient deception, but she stated that in the privatized sector, the patient deception is more prevalent. Therefore, deception is closely tied to doctors’ financial incentives; this is a tragic issue in medicine. 

[*Yeah, exactly, and it is tragic. In the field of women’s studies, you can simply see an endless number of cases. I myself do not dare visit a gynecologist!!!*] (Gynecologist of FGD2)

***Doctors’ Personality***

Distorted DPRs are also related to another factor; the doctor’s personality. How the doctor communicates and how he/she views the patient is a matter of personality, which is both psychological and educational. From this perspective, doctors’ personality has an important function in DPRs and through their unselfish individual character they can establish a more active interaction and the opposite is also true. 

[*I think the most important issue is the individual personality of doctors. Some individuals are not mature enough to respect or value patients’ self-expressions and see the patient as a mechanical object to be given a simple prescription. This is what they believe a DPR should be.*] (Psychologist of FGD2) 

Therefore, the extent to which the doctor thinks about personal and financial benefits or resists suppressing or deceiving the patient is related to his personality and the family setting that he/she was raised in. 

**Paradigm Model**

**Figure 1:**
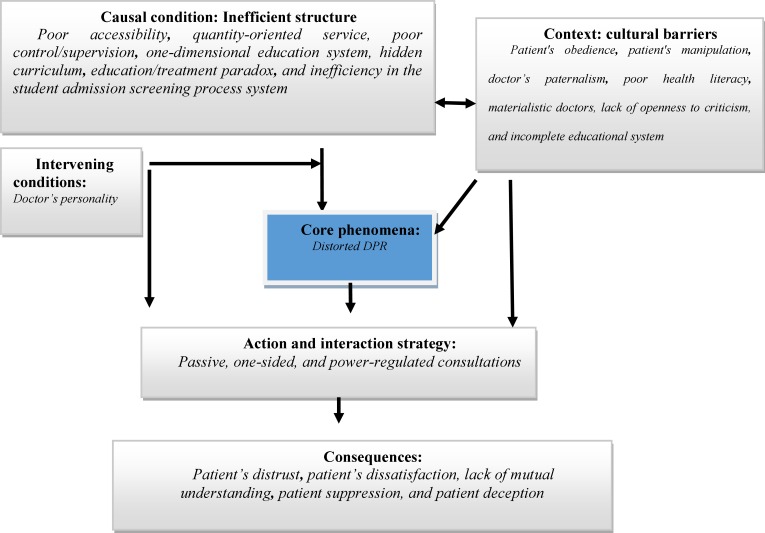
A paradigm model of distorted doctor-patient relationship (DPR)

The paradigm model is a holistic framework that explains social phenomena, specifically the condition which has created them. Such an explanation includes causal conditions, the context, intervening conditions, core phenomenon, action/interaction strategies, and consequences. To reach the paradigm model of this research, the factors observed were conceptually grouped. This was conducted based on data, memos, and the themes that were extracted, and through the establishment of a reflexive manner between the factors and the data. Figure 1 illustrates the paradigm model of distorted DPR. 

As figure 1 illustrates, the core phenomenon in the model is distorted DPR, which is affected by causal conditions (inefficient system), context, and intervening conditions. The inefficient system is a context incapable of meeting patients’ needs and is characterized by inaccessibility, quantity-orientated service, poor control/supervision, one-dimensional education system, education/treatment paradox, and lack of planning. 

In addition to inefficient structure, the context also contributed to the reproduction of distorted DPRs. As the model illustrates, cultural attributes have led to the reproduction of certain values, both in case of doctors and patients. Patients' obedience is accompanied by the exploitation of patients, and doctors’ paternalism, self-interest, and authority.

Such attributes have led to the reproduction of some values in the society, inclining distorted DPRs. The result of these attributes is the reproduction of a value that legitimizes doctors’ unequal and dominance-regulated relationships. For instance, the fact (as confirmed by participants in this study) that the doctor should dominate the relationship was both a tendency in patients’ behaviors and in doctors’ paternalism.

In such conditions, the main question is as to why a patient seeks consultation, when there is no hope for a suitable interaction. It seems that the determining factor is the doctors' personality and not the code of ethics. Thus, this is a double-edged sword that can play either a positive or a negative role. The only reason this type of interaction continues is the hope for a positive interaction that can ultimately lead to deception and a distorted DPR. Hence, when there is a lack of appropriate infrastructure, everything depends on the doctors' personality and character. Based on the results of this study, we can conclude that, since the majority of physicians are financially motivated, it is evident that all DPRs are distorted. 

In this context, action/interaction strategies were passive, one-sided, and power-regulated consultations based on the doctor’s authority. Passive interaction takes place when the doctor does not establish an appropriate interaction and the doctor normally relies on para-clinical data and sometimes physical examination rather than a dialog with the patient. One-sidedness also means that in the interaction the doctor is the final decision-maker. Finally, the consequences of distorted DPR are patient’s distrust**, **patient’s dissatisfaction, lack of mutual understanding, patient suppression, and patient deception. 

## Discussion

The present study revealed that DPR in the context of the study was not suitable which, in this text, was called distorted DPR. This means that there is no communicative relationship between the doctor and patient. Thus, both the doctor and patient, especially the patient, are dissatisfied. The paradigm model revealed that the *inefficient structure* was the main reason behind the formation of distorted DPRs. Poor accessibility, a dominant quantity-oriented approach to providing services, poor control/supervision, and a glaring inconsideration of communicative skills training for doctors and medical students were all the fundamental specifications of this inefficient structure. This shows that the system does not have an effective function, control, and surveillance. Furthermore, cultural values contribute to a distorted DPR. Under such circumstances, personal characteristics of doctors could serve as an intervening factor in generating distorted DPRs. This ultimately means that if a doctor is good, the interaction is also good and vice versa. Thus, DPR is heavily dependent on social and personal character of the doctor rather than systems rational. 

The study by Mishler revealed that doctors and patients had two opposing and unharmonious voices that lead to conflict, and the cessation and disintegration of consultations. It was concluded that there were two voices in DPR; the voice of the life world and that of the system (36, 37). The voice of the life world preferred a technical interpretation of matters, listening, open-ended questions, negotiation, and power distribution. Nevertheless, the voice of doctors was regulated by concentration and preservation of knowledge and asymmetric power relations. In the life world voice of patients, there was a coherent and sensible report, whereas in the voice of doctors, there was suppression, dismantling, and incoherence. Finally, medical care was only effective and humanistic in the life world voice, but in the voice of doctors, it was inhumane and ineffective (38).

Following Mishler’s work, Fairclough dealt with a discursive evaluation of doctors’ tendency to control the interaction. His study showed that doctors used linguistic strategies to control the interaction with the patient (39). The research by Atkinson and Atkinson also showed that doctors did not necessarily use a specific language (medicine) in their interaction with patients, but they used different languages, each of which played a specific function in the interaction (40). 

Barry et al. revealed that the nature of an interaction depends on the type of voice used by doctors and patients. It is also related to the nature of the illness. For example, the worst outcomes occurred when patients used the voice of the life world, but were disregarded (life world disregarded) or blocked (life world blocked) by doctors' use of the voice of medicine (chronic physical complaints) (38). 

A recent critical study was conducted on DPR as a PhD dissertation in the same context under study here (41). The findings of the study revealed that dialogues and DPRs involved a special political economy of medicine which reproduced doctors’ interests within a scientific discourse (14). Furthermore, this confirms our findings that doctors are becoming increasingly materialistic. Another study showed that the medical field was governed by power iniquities in which doctors could finalize their interaction with patients or suppress them whenever they saw fit (6). 

The comparison of findings of the present study to those of the researches reviewed revealed that each of them unfolded a portion of DPR realities, which can be divided into two general subcategories of ontological and structural. Although the mentioned researches mostly revealed ontological and epistemological aspects of DPR, the present study mainly identified the malfunction of the health system structure within the cultural context in case of distorted DPRs. Generally, it can be said that the most important finding in this study is that inefficient infrastructure plays a central role in the continuity of distorted DPR. Under these circumstances, DPR occurs in a chaotic state which is totally dependent on doctor's personality. As was mentioned, better organization of the health care system can limit the number of cases of corruption between doctors and patients (42). In this regard, it was suggested that e-health system should monitor and evaluate the observance of ethics by physicians (43). 

An important limitation of this study was that it was conducted with views of faculty members. However, the views of patients, patients’ relatives, and managers of hospitals and clinics are also important to develop the subject. We suggest that future studies follow the subject with regard to the views of these individuals. Furthermore, further studies with emphasis on quantitative approach are required to evaluate our findings. Moreover, another limitation was that there were no similar studies with which to compare the results of the present study. 

## Conclusion

Our results show that distorted DPR is a natural phenomenon in our context. Even though others have stated that distorted DPR is part of modern medicine, this issue in our context is related to inefficient infrastructure of the health care system. This inefficient infrastructure does not teach doctors social and medical ethics and does not have appropriate supervision over their behavior. Thus, DPR is completely dependent on the individual character of the doctors rather than the rationality of the system. Therefore, this leads to ignorance of patients’ rights which ultimately results in inappropriate DPR and brings about deception, dissatisfaction, and cost burden. This illustrates the necessity of a real change in policymakers’ approach and the way they view the health care system in order to improve its infrastructure. Therefore, medical ethics has to be thought in a way that physicians are socialized ethically, in addition to being controlled and surveyed. 
